# Iodine Content of Commercially Available Iodized Salts in Hungary Determined by Iodometric Titration: Implications for the Effectiveness of Salt Iodization

**DOI:** 10.3390/nu18071164

**Published:** 2026-04-07

**Authors:** Nicole Hunter, Károly Berényi, Ágnes Csergő, Afshin Zand, Anita Bufa, Ágnes Dörnyei, Balázs Németh, István Kiss, Bálint Árvay, Katalin Szendi

**Affiliations:** 1 Institute of Public Health Medicine, Medical School, University of Pécs, 7624 Pécs, Hungary; 2 Institute of Bioanalysis, Medical School, University of Pécs, 7624 Pécs, Hungary; 3 Department of Analytical and Environmental Chemistry, Institute of Chemistry, Faculty of Science, University of Pécs, 7624 Pécs, Hungary

**Keywords:** iodine deficiency, iodized salt, iodometric titration, hypothyroidism

## Abstract

Background/Objectives: Iodine deficiency remains an important global public health concern. Although iodized salt is the primary strategy for iodine deficiency prevention, its effectiveness depends on adequate iodine concentrations in commercially available products. However, laboratory data on the iodine content of retail salt products in Hungary are currently lacking. Therefore, this study aimed to determine the iodine concentration of commercially available iodized table salts in Hungary and to assess their compliance with the WHO-recommended range of 20–40 ppm. Methods: Twenty different brands of iodized table salt were purchased from major retail outlets in Pécs, Hungary, representing the dominant food retail sector. According to product labels, ten salts were fortified with potassium iodate (KIO_3_) and ten with potassium iodide (KI). Iodine concentrations were determined by iodometric titration following WHO-recommended laboratory methods. All measurements were performed in triplicate and expressed as mean values. In addition, a small exploratory wholesale micro-survey examined purchasing patterns of iodized and non-iodized salt in the regional supply chain. Results: Measured iodine concentrations varied substantially among products, ranging from 0 to 33.9 ppm. Overall, 65% of the analyzed salt samples contained less than 20 ppm iodine, while only 35% fell within the WHO-recommended range of 20–40 ppm. Notably, several products declared iodine concentrations below recommended levels on their labels. The wholesale micro-survey showed that ten times more iodized than non-iodized salt was ordered during the observation period. Conclusions: These results suggest that the presence of iodized salt on the market does not necessarily guarantee adequate iodine supply and highlight the potential relevance of considering iodine status during the differential diagnosis of hypothyroidism.

## 1. Introduction

Thyroid disorders represent a major public health concern worldwide and their occurrence is influenced by multiple nutritional, environmental, and demographic factors. Among these, iodine intake plays a central role in thyroid hormone synthesis and population-level thyroid health. Although iodine prophylaxis programs based on salt iodization have been implemented in many countries, questions remain regarding the current effectiveness of these strategies under changing dietary patterns and food production systems.

To provide context for the present study, the Introduction first summarizes the clinical and epidemiological relevance of hypothyroidism, followed by an overview of current knowledge on iodine deficiency globally and within Europe, and finally discusses iodine prophylaxis through iodized salt with reference to Hungary. This framework allows evaluation of whether iodine intake may still contribute to thyroid dysfunction in populations where iodized salt is widely assumed to have eliminated iodine deficiency.

### 1.1. Hypothyroidism

Understanding the epidemiology and clinical significance of hypothyroidism is essential when evaluating potential nutritional contributors to thyroid dysfunction.

Hypothyroidism is a clinical condition characterized by insufficient thyroid hormone production. It typically presents with elevated serum thyroid-stimulating hormone (TSH) levels and is accompanied by reduced circulating free thyroxine (FT4) concentrations [1]. In iodine-deficient regions such as central Asia and Africa, central and eastern Europe, the central U.S., and mountainous regions such as the Alps, Andes, and Himalayas [2], iodine deficiency remains the leading cause of thyroid disorders worldwide. It contributes to goiter development and hypothyroidism in numerous populations [3]. Despite global progress regarding iodine prophylaxis, a considerable proportion of the world’s population, approximately one-third, still lives in regions where iodine intake remains insufficient [3].

Subclinical hypothyroidism is biochemically defined as elevated serum TSH levels as well as the presence of circulating free thyroxine (FT4) concentrations within a set reference range [4]. Subclinical hyperthyroidism is also relatively common and frequently remains undiagnosed in routine clinical care. Additionally, a population’s iodine status may also influence the overall pattern of thyroid dysfunction [5]. A systematic review and meta-analysis published in 2019 estimates that the prevalence of hypothyroidism in European populations (particularly subclinical hypothyroidism) is approximately 3–5%. These estimates most likely reflect improvements in both diagnostic practices as well as differences in iodine status between populations [6].

Publicly available epidemiological data on the prevalence of hypothyroidism (particularly subclinical hypothyroidism) are limited in Hungary. An earlier Hungarian screening study conducted in an elderly population living in an iodine-deficient region of Northern Hungary reported a prevalence of subclinical hypothyroidism of 4.2%, while goiter was detected in 39.4% of the examined individuals [7]. Although this study does not provide long-term epidemiological trends, it represents one of the few domestically available datasets addressing thyroid dysfunction in relation to iodine status. More recent national statistics suggest that the overall burden of thyroid disorders in Hungary has increased over the past decades. According to data from the Hungarian Central Statistical Office, based on general practitioner registry data for adults aged 19 years and older, the number of patients registered with thyroid diseases based on abnormal thyroid function has risen steadily between 1999 and 2023. In 2023, thyroid diseases were reported in nearly 700,000 women (~17% of the adult female population) and slightly more than 100,000 men in Hungary [8]. Unfortunately, this registry data does not distinguish between specific thyroid disorders, nor is it linked to underlying etiology. Therefore, it does not allow for the direct estimation of hypothyroidism or subclinical hypothyroidism prevalence in relation to iodine status. Nonetheless, the data indicates a substantial and growing thyroid disease burden on the Hungarian population and the country’s healthcare providers.

From a clinical perspective, it is understandable why iodine deficiency may remain unrecognized as an etiological factor in patients with hypothyroidism, given that routine diagnostic protocols typically do not include an assessment of one’s iodine status. In confirmed cases of primary hypothyroidism, levothyroxine (LT4) replacement therapy is considered the standard treatment, regardless of the underlying cause. This approach is widely applied in clinical practice, including in Hungary [9,10,11]. However, thyroid hormone replacement therapy may be associated with adverse effects such as tachycardia, palpitations, atrial fibrillation, or worsening of angina [1]. Long-term therapy has also been linked to disturbances in bone metabolism, an increased risk of osteoporosis [12], potential risk of breast cancer [13], as well as neurological and psychological symptoms [10]. In cases where iodine deficiency contributes to thyroid dysfunction, correction of the nutritional deficiency may represent a potential etiological intervention alongside standard clinical management.

These hypotheticals raise an important clinical question: “If iodine deficiency contributes to thyroid dysfunction in certain patients, why is a patient’s iodine status rarely focused on before initiating lifelong hormone replacement therapy?” From a physiological perspective, restoring adequate iodine intake in a patient may allow the thyroid gland to restore endogenous hormone production in cases where iodine deficiency plays a causal role, thereby naturally regulating hormone synthesis according to their body’s needs.

In Hungary, both clinicians and the public often assume that iodine deficiency has largely been irradicated due to the long-standing practice of salt iodization. Consequently, iodine deficiency may not always receive systematic consideration in the differential diagnosis when patients present with suspected hypothyroidism. Assessment of iodine status is generally not included in routine laboratory panels used for the evaluation of thyroid dysfunction, although iodine intake can theoretically be estimated by measuring urinary iodine excretion, particularly in 24-h urine samples [14]. Similar diagnostic approaches are observed in many other countries [15]. Moreover, some laboratories have introduced the measurement of serum iodide concentrations as an alternative indicator; however, serum iodide levels are not considered reliable markers of iodine nutritional status because circulating iodide is rapidly taken up by the thyroid gland or excreted in the urine [16].

### 1.2. Iodine Deficiency

According to the World Health Organization (WHO), the recommended daily iodine intake is 120 µg for school-aged children, 150 µg for adults, and approximately 200 µg for pregnant and lactating women [17]. Because iodine intake is a key determinant of thyroid hormone synthesis, the population burden of hypothyroidism is closely linked to the iodine status of a given region.

Despite decades of prevention programs, iodine deficiency continues to represent an important public health concern worldwide [18]. According to the 2025 Global Scorecard of Iodine Nutrition published by the Iodine Global Network (IGN) [19], which compiles data primarily from urinary iodine measurements in school-aged children after 2009, several European countries remain iodine insufficient. Countries include Finland, Germany, Liechtenstein, Lithuania, Norway, Slovenia, Sweden and Ukraine. For a few European countries, including Hungary, recent nationally representative data is not available. The WHO has also emphasized that iodine deficiency, particularly mild deficiency, remains a persistent nutritional problem in the WHO European Region despite previous improvements in iodine nutrition [20]. Therefore, continued monitoring of iodine status remains necessary even in developed countries, especially across Europe. European monitoring data indicates that approximately 44% of school-aged children may have insufficient iodine intake [21]. In Hungary, the most recent population-based urinary iodine assessment among school-aged children (10–14 years) is from 2005, which suggested adequate iodine status in the surveyed locations [19,22]. However, the survey was conducted in only three regions, and the authors themselves noted that the results may not necessarily be representative of the entire country.

Earlier studies conducted in Hungary consistently suggest insufficient iodine intake in several population groups. A nationwide investigation among schoolchildren performed between 1994 and 1997 reported iodine deficiency in multiple Hungarian regions [23]. Szabolcs et al. found that elderly populations living in iodine-deficient regions also showed inadequate iodine intake and evidence of iodine deficiency [7]. Studies focusing on pregnant women have also indicated suboptimal iodine status among Hungarians. Mezősi et al. found that a high proportion of urine samples indicated iodine deficiency during pregnancy, while iodine excretion in non-pregnant women was also below optimal levels (median 82 µg iodine/g creatinine) [24]. Interestingly though, iodine status remained insufficient (median 68 µg iodine/g creatinine) even among women consuming iodized salt. This suggests that iodized salt alone does not ensure adequate iodine intake during pregnancy. Additional regional studies have produced similar findings. Investigations in Northern Pest and Western Nógrád counties reported urinary iodine levels below 100 µg/L in nearly half of pregnant women, with more than 10% showing values indicative of severe iodine deficiency (<20 µg/L) [25]. Recent Hungarian studies also suggest suboptimal iodine intake in newborns. Research examining neonatal iodine metabolism in Hajdú-Bihar County found that only about 30% of newborns had urinary iodine concentrations consistent with adequate iodine supply, while a substantial proportion showed low values (<50 µg/L) [26]. Similarly, a study investigating iodine status during pregnancy and lactation reported iodine deficiency in approximately 85% of samples. Even among women using iodized salt, urinary iodine excretion remained low (46.7 µg iodine/g creatinine), and iodine supplementation resulted in only moderate improvements (55.1 µg iodine/g creatinine) [27]. Additionally, Katkó et al. reported reduced iodine excretion in pregnant women who did not receive iodine supplementation and in those relying solely on iodized salt intake [28].

Considering the above-mentioned points, it is evident that recent and up-to-date iodine status data is lacking in Hungary, while earlier studies across different population groups, including schoolchildren, pregnant women, and newborns, have repeatedly indicated insufficient iodine intake.

### 1.3. Iodine Prophylaxis and the Use of Iodized Salt

Given the central role of iodine deficiency in thyroid dysfunction, public health strategies have focused primarily on improving iodine intake at the population level.

Universal salt iodization (USI) remains the cornerstone of iodine deficiency prevention. A typical salt iodization protocol assumes that approximately 20% of iodine will be lost between production and household storage, and an additional 20% will be lost during cooking. WHO/UNICEF/ICCIDD guidelines have historically recommended iodine concentrations of roughly 20–40 mg iodine per kg of salt (20–40 ppm) at time of production to deliver around 150 µg iodine per person per day. This protocol assumes that one’s average salt intake is about 10 g/day [20,29,30]. For monitoring purposes, WHO indicators commonly define “adequately iodized” household salts as containing 15–40 ppm iodine, and USI programs generally target high household coverage (often >90%) [31]. However, these long-standing assumptions warrant re-evaluation given changing diets and public health initiatives to reduce salt. Average salt consumption varies significantly across countries and has been on the decline due to policies aimed at reducing salt intake (e.g., toward ~5 g/day) [32]. As a result, optimal iodine concentration targets, which were based on people having a higher salt intake, may not align with current consumption patterns, particularly when a large portion of one’s salt intake originates from foods prepared outside the home.

Recent global household data suggests that the use of iodized salt is widespread, yet coverage and the adequacy of those salts vary considerably between countries. Estimates made by UNICEF indicated that about 89% of the global population consumed salt with some form of iodine in 2020, while also highlighting that insufficient data was available to generate estimates for several regions, including parts of Europe and North America (2014–2020) [33]. WHO guidelines emphasize that rapid test kits are suitable for qualitative screening, even though many rapid test kits can merely confirm the presence of iodine rather than provide accurate quantification [30]. Iodometric titration (or equivalent quantitative methods) is required to determine iodine concentration reliably. Therefore, rapid-test-only surveys can only estimate the proportion of households using iodized salt but cannot determine whether the salt is adequately iodized (≥15 ppm).

The WHO has concluded that adequate population iodine status is most reliably achieved in countries where iodized salt is used not only at the household level but also in processed foods [20]. This is particularly important given that approximately 70–80% of total salt intake originates from processed or commercially prepared foods in a modern diet rather than from salt added during home cooking [34]. Consequently, the effectiveness of iodine prophylaxis depends on the increased use of iodized salt by the food industry. Within the WHO European Region, 20 Member States and Kosovo have implemented mandatory salt iodization policies requiring that nearly all foods containing salt be produced using iodized salt [20]. However, in 24 countries, the use of iodized salt in processed foods remains voluntary or unregulated. As a result, commonly consumed food products are often manufactured with non-iodized salt. A recent market survey highlighted this shift with only 9% of salt used in processed foods in Germany and 34% in Switzerland was found to be iodized [34].

The effectiveness of Hungary’s iodine prophylaxis at a population level is constrained by limited and selective monitoring data. While the most recent available population-based urinary iodine assessment in school-aged children dates to 2005, which suggested adequate iodine status in the surveyed locations at the time, this study was conducted in only three regions and thus cannot be generalized to the full population [19,22]. Hungarian public catering regulations mandate the use of iodized salt in meal preparation in institutional settings (e.g., schools, kindergartens, health and social care facilities). Specifically, the national decree 37/2014 (IV.30.) EMMI, §12(2) requires that food preparation in public catering use exclusively iodized table salt compliant with the MSZ-01-10007 standard (equivalent to ISO 10007) [35]. This regulatory environment makes it plausible that school-aged children may be exposed to iodized salt more consistently than adults, potentially contributing to better iodine status among children than in adult or pregnant populations. This phenomenon is also discussed in the broader WHO European context [20]. This situation may also reflect what has been described as “silent iodine prophylaxis” in Hungary. According to Péter (2020) [36], despite the absence of a fully regulated universal iodization program, public health awareness campaigns and voluntary use of iodized salt have likely contributed to improvements in population iodine status over past decades. However, such an approach may only partially correct iodine deficiency, as it relies on voluntary consumption patterns and industrial use. For effective elimination of iodine deficiency, the author argues that iodized salt should replace non-iodized salt in nearly all food products, including those produced by the food industry.

Despite the long-standing reliance on iodized salt as the primary preventive strategy, no published Hungarian laboratory studies (to our knowledge) have systematically quantified the iodine concentration of commercially available iodized table salts using quantitative analytical methods. This research gap is relevant given that USI program effectiveness depends on whether retail products comply with target specifications at the time of use, not simply on their labelled iodized status.

Quantitative determination of iodine in salt is typically performed using iodometric titration, which is considered a reference method in WHO/UNICEF/ICCIDD guidelines, although alternative techniques such as potentiometry or spectrophotometry have also been applied in research settings [30].

Several studies from different regions [37,38,39,40,41,42,43,44,45,46,47,48,49] have directly measured iodine concentrations in commercially available iodized salts, reporting substantial variability across markets. While some countries demonstrate relatively high compliance with recommended iodization levels [37,38,41,43,44,46,48,49], others report a considerable proportion of samples containing iodine concentrations below the WHO-recommended range of 20–40 ppm [39,40,42,45,47]. These findings suggest that the presence of iodized salt on the market does not necessarily guarantee adequate iodine content at the point of use.

The aim of the present study was to evaluate the iodine content of commercially available iodized table salts in Hungary. Specifically, we sought to (1) determine iodine concentrations in products obtained from the dominant retail sector, (2) assess compliance with the recommended 20–40 ppm iodine specification at the point of use, and (3) provide evidence that may support the refinement of national iodine supplementation and diagnostic strategies. In addition, a micro-survey was conducted to provide contextual insight into iodized versus non-iodized salt distribution in the food service sector.

## 2. Materials and Methods

### 2.1. Salt Samples

On 16 February 2026, a total of 20 different brands of iodized table salt were purchased from retail outlets in the city of Pécs, Hungary. Retail sampling covered the dominant food retail sector, including hypermarkets, discount chains, wholesale suppliers and international retail chain stores widely present across Europe, encompassing 11 major retail outlets representing the mainstream food distribution network. Among the collected samples, 10 salts were fortified with potassium iodate (KIO_3_) and 10 with potassium iodide (KI), according to the information provided on the product labels. The labelled characteristics of the purchased salt products are summarized in Table 1. One product (#20) did not contain added iodine; however, this type of non-iodized rock salt has been reported to contain trace amounts of naturally occurring iodine.

Information provided on product labels indicated certain Hungarian companies as manufacturers. However, according to publicly available information on the companies’ websites, some of these entities primarily perform packaging, storage, and distribution activities rather than salt production or iodization. For example, Só-Center Packaging and Trade Social Cooperative (Nyírmada, Hungary) is listed on several product labels as the manufacturer, while the company’s own description indicates that it focuses on packaging, trade, and warehousing. Similarly, Solinwest 2000 Ltd. is also identified as a manufacturer on product labels, although company information suggests that it primarily operates as a distributor and supplier. Based on the information available on the product labels and on the websites of the companies listed as manufacturers, no evidence was found that iodization of salt is performed in Hungary by these entities. In addition, several companies were contacted by email to clarify their role in the production chain; according to the response received from a company representative familiar with the Hungarian salt market, iodization of table salt is not carried out in Hungary.

### 2.2. Measurement Method, Iodometric Titration

The iodine content of the iodized salt samples was determined by iodometric titration following the WHO/UNICEF/ICCIDD recommended laboratory methods for iodine determination in salt [30]. Prior to analysis, the unopened salt packages were stored for several days in the dark at room temperature. Measurements were performed immediately after opening the packages to minimize potential iodine loss. All measurements were performed in triplicate.

#### 2.2.1. Reagents and Chemicals

All reagents were of analytical grade and used as received. Potassium iodate (KIO_3_) was obtained from Reanal (Budapest, Hungary). Potassium iodide (KI) was purchased from Molar Chemicals (Halásztelek, Hungary). Sodium thiosulfate (Na_2_S_2_O_3_, anhydrous, ≥99%) was obtained from Thermo Scientific (Waltham, MA, USA). Sulfuric acid (H_2_SO_4_, 96%, RPE grade) was supplied by Carlo Erba Reagents (Milan, Italy). A commercial sodium hypochlorite solution (NaOCl, approximately 40 g/L active chlorine) was used as the oxidizing agent and obtained from Chemitat Ltd. (Szeged, Hungary). Soluble starch was obtained from a pharmaceutical supplier (University Pharmacy, Pécs, Hungary).

#### 2.2.2. Calculation of Iodine Content

The iodine content of the salt samples fortified with potassium iodate (expressed as ppm or mg/kg) was calculated from the measured sodium thiosulfate consumption using the following formula:

Iodine content (ppm) = Titrant consumption (mL) × Thiosulfate factor × 0.10575 × 25

where 0.10575 represents the mass of iodine corresponding to the amount of potassium iodate reduced by 1 mL of 0.005 N sodium thiosulfate, and the factor 25 accounts for the conversion from the analyzed 40 g salt sample to 1 kg of salt.

The same calculation approach was applied for salts fortified with potassium iodide.

#### 2.2.3. Procedure for the Determination of Potassium Iodide (KI)

##### Testing for the Presence of Stabilizers (Reducing Agents)

For most iodide fortified salts (KI), the packaging did not indicate the presence of a stabilizing agent (except for samples #16 and #19). Nevertheless, all KI fortified salts were tested to determine whether they contained reducing agents that could affect the formation of free iodine and thereby interfere with the iodometric determination.

For this purpose, 40 g of KI fortified table salt was dissolved in 200 mL of bi-distilled water. Subsequently, 2 mL of the KIO_3_ stock solution and 1 mL of 0.8% starch indicator solution were added. While continuously stirring, 5 mL of sulfuric acid was then added gradually to the solution. Upon addition of the first milli-liter of sulfuric acid, a blue color reaction appeared, indicating the formation of an iodine–starch complex. However, the blue color disappeared spontaneously within approximately 5–15 s. Further addition of acid did not influence this observation.

Based on these results, it was concluded that despite the absence of labeling, all KI fortified salts likely contained a reducing agent capable of reducing free iodine, most probably sodium thiosulfate or sulfite. This phenomenon was also observed for sample #20, which according to the food label did not contain added iodine but was described as containing iodine from natural sources.

##### Titration Procedure

The titration procedure was based on the WHO recommended iodometric method for iodine determination in salt [30], with minor modifications described below.

For the determination of iodine content, 40 g of salt was dissolved in 300 mL of bi-distilled water. Subsequently, 7 mL of 1 M sulfuric acid was added, followed by 1 mL of 5% hypochlorous acid solution (commercial sodium hypochlorite, “bleach”) under a fume hood. During continuous stirring, the solution developed a yellowish color and the characteristic pungent odor of chlorine gas, indicating the formation of free chlorine. To ensure completion of the reaction, the solution was stirred for 5 min and then boiled vigorously for one hour to remove excess chlorine gas. During boiling, bi-distilled water was added as needed to compensate for evaporative losses, ensuring that the solution volume did not decrease below 200 mL to prevent salt precipitation upon cooling. After cooling, the solution was processed according to the previously described KIO_3_ determination procedure. Briefly, 0.5 g of solid KI was added under continuous stirring (the acidic pH was verified using pH indicator paper), and the mixture was kept in the dark for 10 min to allow complete liberation of iodine. The liberated iodine was then titrated with the sodium thiosulfate solution. Near the endpoint, starch indicator solution was added, and the titration continued until the disappearance of the blue color.

##### Method Testing and Optimization for Potassium Iodide (KI) Determination

To evaluate and optimize the analytical procedure, a potassium iodide (KI) stock solution was prepared by dissolving 0.345 g KI in 100 mL of bidistilled water (3.45 mg/mL). From this solution, 0.500 mL was transferred into an Erlenmeyer flask containing 300 mL of bidistilled water. Subsequently, 7 mL of sulfuric acid and 1 mL of 5% sodium hypochlorite solution (“hypo”) were added. To ensure completion of the reaction, the solution was stirred for 5 min, and the mixture was stirred, boiled, and then allowed to cool. Based on pH measurements, the solution had a pH of approximately 2 (or slightly below), and therefore no additional acidification was considered necessary. After cooling, 0.5 g of solid KI was added under continuous stirring, and the solution was kept in a closed container in the dark for 5 min. The liberated iodine was then titrated with sodium thiosulfate solution until close to the endpoint, after which starch indicator was added and the titration continued until the endpoint was reached. Based on the theoretical calculations, a titrant consumption of 12.5 mL was expected. This value was derived from the complete oxidation of KI (3.45 mg/mL) to iodate, corresponding to 0.0104 mol KIO_3_, which was calculated based on the stoichiometry of the iodometric reaction with KI and the 0.005 N sodium thiosulfate solution. The method was evaluated in triplicate under several experimental configurations.

During the optimization phase, experiments were performed using the 0.500 mL KI stock solution with the addition of either 2 mL or 5 mL of 5% sodium hypochlorite solution. With 1 mL of sodium hypochlorite, the chlorine odor disappeared completely after approximately 40 min of boiling, whereas with 5 mL of sodium hypochlorite more than 1 h of boiling was required. Increasing the sodium hypochlorite concentration did not affect recovery accuracy.

To simulate the presence of reducing agents that may occur in commercial iodized salts, the same experimental setup was repeated with the addition of 15 mL of 0.005 N sodium thiosulfate to the 300 mL bidistilled water before the KI stock solution (0.500 mL), sulfuric acid (7 mL), and sodium hypochlorite. In these experiments, the addition of 1 mL sodium hypochlorite was also sufficient for complete oxidation.

The experiment was further repeated using a salt matrix prepared by dissolving 40 g of pharmaceutical-grade NaCl in 300 mL of water. In this setup, 15 mL of 0.005 N sodium thiosulfate was added to simulate the presence of stabilizing agents in iodized salts, followed by the addition of 0.500 mL KI stock solution, sulfuric acid, and sodium hypochlorite. A measurement series was then conducted using different volumes of the KI stock solution (200 µL, 500 µL, and 700 µL). The method demonstrated good linearity and recovery. The expected sodium thiosulfate consumptions of approximately 5.0 mL, 12.5 mL, and 17.5 mL were recovered with deviations of approximately 0.1 mL.

Based on the results of the initial experiments with commercial salts, a boiling time of at least 60 min was selected for routine measurements. In some sea salt samples, chlorine removal occurred more slowly than in solutions containing pure NaCl.

Additional recovery tests were performed in three salt samples by spiking the solutions with an additional 200 µL of KI stock solution.

### 2.3. Validation

The validation of the analytical procedures for the determination of KIO_3_ and KI is provided in Supplementary Materials.

### 2.4. Wholesale Micro-Survey

Purchase data covering a three-month period (June–August 2025) were obtained from a wholesale distributor located in Baranya County, Hungary. The distributor, a major supplier in the region, provided information on the quantities of iodized and non-iodized salt ordered during this period and on the types of institutions placing these orders.

## 3. Results

### 3.1. Iodometric Titration

Table 2 presents the iodine content declared on the product labels and the iodine concentrations measured by iodometric titration for the 20 salt samples analyzed in this study.

**Figure 1:**
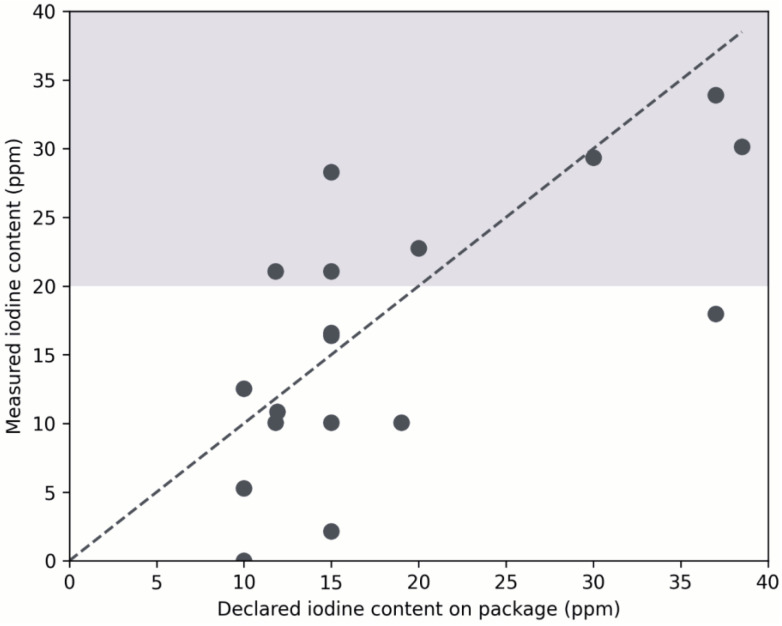
Declared versus measured iodine concentrations in commercially available iodized salts.

At the retail level, iodine concentrations within the WHO-recommended range for iodized salt (20–40 ppm) were found in seven samples. Four samples were fortified with potassium iodate (KIO_3_) and three with potassium iodide (KI) (#2, #7, #8, #9 and #11, #14, #16, respectively) (Figure 1). Overall, categorization of the measured iodine concentrations showed that 65% of the analyzed salt samples contained less than 20 ppm iodine, while only 35% fell within the WHO-recommended range of 20–40 ppm (Table 3). No samples exceeded the upper limit of the recommended iodine range.

For two potassium iodide fortified salts (#12 and #14), the same manufacturer was indicated on the product label (KALAS S.A., Athens, Greece); however, different iodine concentrations were measured in the two products. A similar observation was made for three potassium iodate fortified salts (#1, #3 and #10), for which the same company (Só-Center Packaging and Trade Social Cooperative, Nyírmada, Hungary) was indicated as the manufacturer on the product labels. Despite this, iodine concentrations differed substantially among these samples, with one product showing no detectable iodine (0 ppm). As discussed in Section 2, this company appears to perform packaging and distribution activities rather than salt production or iodization.

### 3.2. Iodine Content Declared on the Packaging

Regarding the iodine content declared on product labels, seven of the ten potassium iodate (KIO_3_) fortified salt brands indicated iodine concentrations below the WHO-recommended range of 20–40 ppm elemental iodine, while in one case the iodine content was not specified on the packaging. Among the 10 potassium iodide (KI) fortified salt brands, six declared iodine concentrations below the recommended range, and in one case, the iodine content was not indicated on the label. In addition, one product (#12) did not specify either iodide or iodate as the iodine compound on the packaging. However, subsequent analysis revealed the presence of iodide. Furthermore, six salt brands (#1, #3, #5, #6, #7 and #10) declared iodine concentrations below 15 ppm, which is the lower threshold commonly used to define adequately iodized salt at the household level (Figure 1). Notably, this pattern was observed only among salts fortified with potassium iodate (KIO_3_). Figure 1 illustrates the relationship between the iodine content declared on product labels and the concentrations measured by iodometric titration in the analyzed salt samples. The diagonal line indicates equality between declared and measured iodine content, while the horizontal line marks the WHO lower recommended limit of 20 ppm. Seven samples exceeded this threshold, while the majority contained lower iodine concentrations.

### 3.3. Presence of Stabilizers in KI Fortified Salts

Among the KI fortified salt products, only two brands explicitly indicated the presence of stabilizers on the packaging (#16: sodium carbonate and magnesium carbonate; #19: sodium sulphate). Stabilizers are commonly added to iodide fortified salts to inhibit the oxidation of iodide (I^−^) to molecular iodine (I_2_), thereby reducing iodide losses and helping maintain the declared iodine concentration during storage. For the remaining KI fortified salt products, no stabilizers were listed on the packaging and the specific reducing agents used for iodide stabilization were not indicated. However, the analytical results suggested that stabilization mechanisms were likely present in these products as well. Unexpectedly, the presence of a stabilizing (reducing) compound was also indicated in the non-iodized salt sample (#20), a naturally occurring rock salt containing only trace levels of iodine.

### 3.4. Wholesale Micro-Survey

To complement laboratory findings a three-month record-based survey was conducted using purchase data from a wholesale distributor. The analysis examined whether customers ordered iodized or non-iodized salt. Orders originated from the retail sector (*n* = 2), commercial food service establishments (*n* = 32) (including restaurants, bistros, pizzerias, hotel kitchens, and small local eateries), institutional catering facilities (*n* = 22) (including schools, hospitals, nursing homes, and camp kitchens), and one food manufacturing plant (*n* = 1). During the three-month period, a total of 7314 kg (~7 tons) of iodized salt and 774 kg (~0.7 tons) of non-iodized salt were ordered from the wholesale distributor.

## 4. Discussion

To our knowledge, no peer-reviewed Hungarian studies have previously quantified the iodine concentration of commercially available iodized table salts using analytical laboratory methods. Given the limited sample size and single-city sampling frame, the findings should be interpreted as indicative rather than representative of the Hungarian market as a whole.

### 4.1. Iodometric Titration

A comparable pattern was previously reported in Hungary by the National Food Chain Safety Office (NÉBIH) in a market survey conducted in 2015, although these results were not published in a peer-reviewed scientific journal [50]. In that survey, 28 different salt products available on the Hungarian retail market were analyzed. The survey found that iodine was not detectable in two samples, and only eight contained iodine concentrations exceeding 20 ppm.

These findings, together with the results of the present study, suggest that the iodine supply provided by iodized table salt available on the Hungarian retail market may be insufficient to reliably support recommended iodine intake levels under real-world conditions, although this cannot be confirmed without population-level iodine status data. Notably, this conclusion arises directly from the analytical results. Even under the simplifying assumption that only iodized salt is consumed, most of the products analyzed in the present study contained iodine concentrations below the WHO-recommended range. In practice, however, iodine intake from salt may be even lower. Non-iodized salt products remain widely available in the Hungarian retail markets, and the proportion of households that regularly use iodized salt is currently not well documented. If a portion of the population preferentially purchases non-iodized salt or does not specifically consider iodization when choosing salt products, the effective dietary iodine intake from salt could be even further reduced. An additional factor that may influence iodine availability is the chemical form used for salt iodization. In Hungary, both potassium iodate (KIO_3_) and potassium iodide (KI) are permitted. While iodate is generally regarded as the more stable compound, iodide is more susceptible to oxidative loss during storage, particularly under unfavorable environmental conditions such as humidity or prolonged storage. Even in the presence of stabilizing agents, gradual iodine losses may occur over time, which could further decrease the effective iodine content of iodized salt at the point of consumption. Interestingly, several of the lowest measured iodine concentrations were observed in salts labeled as potassium iodate (KIO_3_) fortified products, despite iodate generally being considered the more stable iodine compound for salt iodization.

These considerations raise the broader question of how consistently iodized salt products meet regulatory iodine specifications in other countries and whether iodine prophylaxis programs are more strictly regulated or monitored in different settings.

Globally, numerous studies have evaluated iodine concentrations in iodized salt collected at both retail and household levels. In several countries, iodine concentrations in commercial table salts have been measured directly using iodometric titration or comparable analytical techniques. Reported iodine levels vary widely between countries and markets, reflecting differences in iodization practices, regulatory frameworks, and quality control systems. In the following section, selected studies from both low-, middle-, and high-income settings across different regions are briefly reviewed to place the present findings in an international context.

In Sri Lanka, Vithanage et al. reported relatively high iodine concentrations (>30 ppm) in all analyzed samples (*n* = 6). Interestingly, both iodide and iodate were detected in the same salts [37].

In Bangladesh, where iodization of salt for human consumption is mandatory under the Iodized Salt Bill (2021), Islam et al. reported that seven of ten analyzed salt samples contained iodine concentrations within the WHO-recommended 20–40 ppm range [38]. Similarly, a large study by Abdurrahim et al. analyzing 3600 samples found that approximately half of the retail salt samples fell within the recommended range [39].

In Ethiopia, iodization of salt for human consumption has been mandated since February 2011, when the Council of Ministers introduced legislation requiring that all salt intended for human consumption be iodized and comply with national iodization standards. Despite this regulatory framework, reported iodine concentrations in commercially available salts have been variable. Deresa et al. found that four out of six iodized salt samples contained iodine concentrations below the WHO-recommended level (<20 ppm) [40], while Shawel et al. reported that all samples (*n* = 7) collected at the retail level contained ≥20 ppm iodine [41]. Household surveys in Ethiopia also showed substantial inadequacy: Desta et al. reported that 82.6% of household salt samples (*n* = 292) contained <15 ppm iodine and 18.2% contained no iodine at all [42], whereas Anteneh et al. found that 57.4% of household salt samples (*n* = 1194) were adequately iodized [43].

In Liberia, Simbo et al. reported iodine concentrations of 22 ppm and 26 ppm in two brands of iodized salt, although variability within brands could not be assessed due to identical reported values for all sub-samples [44].

Studies from the Middle East and Europe also show considerable variation.

In Jordan, Mahmoud et al. observed that although some marketed salts met iodization standards, the majority did not [45].

In Saudi Arabia, Al-Dakheel et al. reported that 95.2% of household iodized salt samples (*n* = 775) contained ≥15 ppm iodine, with a mean iodine concentration of 51 ppm [46].

Evidence from European settings also indicates substantial variability in iodine content of iodized salt. In Serbia, Rajković analyzed seven commercially available iodized table salts purchased from supermarkets, all of which were labeled as potassium iodide (KI) fortified salts [47]. Three of the seven samples contained iodine concentrations within the 20–40 ppm range. Salt iodization is mandatory in Serbia according to the Regulations of Table Salt Quality Meant for Human Diet [51], which stipulate iodization of salt intended for human consumption with potassium iodide at a level corresponding to approximately 12–18 ppm iodine.

In Turkey, Bilgin et al. analyzed 26 iodized table salts purchased from markets and district bazaars in Istanbul [48]. All analyzed products were labeled as potassium iodate (KIO_3_) fortified salts, and most samples contained iodine concentrations within or above the WHO-recommended range, with only six samples containing <20 ppm iodine. Salt iodization is mandatory in Turkey according to the Turkish Food Codex Communiqué on Salt (No. 2013/48), which requires the addition of potassium iodate at 25–40 mg/kg or potassium iodide at 50–70 mg/kg. However, salts fortified with potassium iodide were not analyzed in Bilgin et al.’s study due to methodological limitations of the iodometric titration approach used.

Similarly, in Romania, Brezean et al. analyzed 102 commercially available table salt samples originating from various European countries and reported that 69% contained iodine concentrations consistent with national legislation [49].

More broadly, studies across Europe have highlighted considerable heterogeneity in iodization practices and iodine content in salt, even in high-income settings, reflecting inconsistencies in regulation, implementation, and quality control [52].

Taking all of this into account, these international studies demonstrate that iodine concentrations in commercially available iodized salt vary substantially across countries and regulatory environments. While some settings report relatively high compliance with recommended iodization levels, others show considerable deviations from national or international standards. Importantly, such variability is not restricted to low- and middle-income countries but is also observed in high-income and European settings, indicating that inconsistencies in iodization practices and quality control represent a global challenge rather than a region-specific issue.

In this context, the results of this study indicate that the proportion of adequately iodized salt products available at the retail level in Hungary may be relatively low compared with several other reported settings.

According to the World Health Organization, in most countries of the WHO European Region, iodized salt is produced in large industrial facilities operating under standardized quality management and food safety systems such as ISO 9001 [53], ISO 22000 [54], Hazard Analysis and Critical Control Points (HACCP), or Good Manufacturing Practices [20]. Despite this expectation of consistent production standards, studies conducted in several European countries have reported heterogeneous iodine concentrations in commercially available salts. For example, investigations from Romania, Serbia, and Turkey have demonstrated that while a proportion of marketed salts comply with national iodization standards, others contain iodine concentrations outside recommended ranges. In line with these observations, the present study also found substantial variability in iodine concentrations among commercially available iodized salts, including differences between products originating from the same manufacturer. Notably, apart from one non-iodized rock salt originating from Pakistan, all analyzed iodized salt products available on the Hungarian market were imported from European countries (Table 1), indicating that such variability may occur even within the European supply chain.

### 4.2. Iodine Content Declared on the Packaging

Importantly, this section refers to iodine concentrations declared on product labels rather than analytically measured values. These declared values reflect manufacturer-defined iodization targets and therefore cannot be attributed to iodine losses occurring during storage, transport, or household use. Consequently, the observation that many products specify iodine concentrations below the WHO-recommended range of 20–40 ppm suggests that, in several cases, iodization levels may be set below internationally recommended targets at the production level. In contrast, measured iodine concentrations (Section 3.1) may be influenced by post-production losses along the supply chain.

Only one quarter of the analyzed salt brands declared iodine concentrations within the WHO-recommended range of 20–40 ppm on the product labels. Several products reported iodine contents around 10–15 ppm, indicating that these salts were not intended to reach the WHO-recommended iodization level even at the time of production. Consequently, the low iodine concentrations observed in many samples may reflect not only iodine losses during storage or distribution but also the iodization targets applied during manufacturing.

One possible explanation is the frequent use of the WHO monitoring indicator defining adequately iodized household salt as containing ≥15 ppm iodine [31]. However, this threshold refers to iodine concentrations measured at the household level after storage and handling under variable conditions, rather than to the iodine concentration at the time of production. Therefore, if iodization targets are set close to this monitoring threshold, the resulting iodine concentration at the point of consumption may fall substantially below the levels required to ensure adequate dietary iodine intake.

### 4.3. Presence of Stabilizers in KI Fortified Salts

In potassium iodide fortified salts, stabilizing agents such as dextrose or sodium thiosulfate are commonly added to inhibit the oxidation of iodide (I^−^) to elemental iodine (I_2_), thereby reducing iodine losses during storage [55]. Although stabilizers can substantially improve the stability of potassium iodide fortified salts [56], potassium iodate (KIO_3_) is generally considered chemically more stable during storage and distribution, particularly under conditions of elevated humidity, temperature, or prolonged storage [57]. For this reason, WHO/UNICEF/ICCIDD guidelines recommend potassium iodate as the preferred compound for salt iodization [30]. Nevertheless, both potassium iodate (KIO_3_) and potassium iodide (KI) are permitted for salt iodization in Hungary, and KI fortified salts represent a substantial proportion of the products available on the retail market.

An additional practical consideration is that product labels typically indicate only that the salt is iodized, while the presence and type of stabilizing agents may not always be specified. As a result, information relevant to iodine stability during storage may not be fully transparent to consumers or researchers.

### 4.4. Wholesale Micro-Survey

In addition to laboratory analysis, a small wholesale micro-survey was conducted to gain exploratory insight into salt purchasing patterns in the regional supply chain. Over the three-month observation period, the wholesale distributor reported orders totaling 7314 kg (~7 tons) of iodized salt compared with 774 kg (~0.7 tons) of non-iodized salt. This approximately ten-fold difference suggests that iodized salt may predominate within the supply network of the studied distributor, including commercial food service establishments, institutional catering facilities, and retail outlets.

From a public health perspective, this finding may indicate that iodized salt is the dominant product within the professional food supply chain in the studied region, which is consistent with the intended goals of salt iodization programs. However, the continued presence of non-iodized salt orders, even if relatively small in volume, suggests that non-iodized alternatives remain available and are still used by some institutions. From a public health perspective, this observation may provide preliminary insight into supply patterns; however, given the exploratory nature of the micro-survey, it should be interpreted with caution. Further investigation in larger and more representative settings would be required to evaluate purchasing patterns and their potential relevance for iodine prophylaxis strategies.

These findings suggest that the widespread availability and use of iodized salt do not necessarily guarantee adequate iodine intake, if the iodine content of the products falls below recommended levels. Consequently, monitoring iodine concentrations in commercially available salts may represent an important complementary component of iodine prophylaxis programs, in addition to monitoring population iodine status.

In this context, insufficient iodine intake may warrant consideration in certain clinical situations, although the present study does not provide direct evidence for clinical outcomes.

Importantly, the present study evaluates iodine content in salt products rather than population iodine status. These findings should therefore be interpreted as indicative of potential limitations in iodine supply rather than direct evidence of inadequate iodine intake at the population level.

### 4.5. Limitations

At the population level, iodine nutrition is typically evaluated using indicators such as household coverage of adequately iodized salt and urinary iodine concentration (UIC). The present study did not assess population iodine status using these established indicators; therefore, our findings cannot be used to directly estimate iodine deficiency prevalence in Hungary.

Second, the sampling frame was restricted to retail outlets in a single city (Pécs) and to a limited number of products (*n* = 20). Although the sampling strategy targeted the dominant retail sector, the results may not fully represent the national retail market, regional supply chains, or seasonal variation in product availability and turnover.

Third, measurements were performed on newly opened packages and reflect iodine concentrations at the time of analysis. We did not systematically evaluate iodine losses under controlled storage conditions, nor could we reconstruct storage duration and environmental exposure (e.g., humidity, temperature, light) prior to purchase. Consequently, the relative contribution of under-iodization at production versus post-production iodine losses cannot be determined from these data alone.

Fourth, while label information was recorded, manufacturer and production-chain attribution were limited by incomplete or inconsistent labeling. In addition, although the presence of reducing agents consistent with stabilizers in KI fortified salts was demonstrated experimentally, the specific compounds used and their concentrations were not analytically identified. Consequently, their precise contribution to iodine stability could not be quantitatively assessed.

Finally, the wholesale micro-survey was based on a single distributor and a limited observation period, and it should be interpreted as exploratory rather than representative of national purchasing patterns.

Despite these limitations, the observed variability and frequently low iodine concentrations in commercially available salts highlight a potential gap between the nominal availability of iodized salt and the effective iodine supply at the point of consumption, although this observation requires confirmation in larger, nationally representative studies.

These limitations should be considered when interpreting the findings and particularly when extrapolating the results to the national level or to clinical contexts. Furthermore, no direct conclusions can be drawn regarding population iodine status or thyroid disease burden based on the present data.

## 5. Conclusions

The present findings suggest that the effectiveness of iodized salt-based iodine prophylaxis in Hungary warrants closer evaluation. Although iodized salt clearly predominated in the regional wholesale supply chain, most commercially available products analyzed in this study contained iodine concentrations below the WHO-recommended range of 20–40 ppm at the retail level. In addition, iodine concentrations declared on product labels were frequently below the recommended range, indicating that some salts may already be under-iodized at the time of production rather than losing iodine solely during storage or distribution. These observations highlight a potential discrepancy between the nominal availability of iodized salt and the actual iodine supply provided by these products.

Several factors may contribute to this situation, including heterogeneous iodization practices across manufacturers, the continued availability of non-iodized salt, incomplete use of iodized salt in the food industry, and broader dietary changes such as salt reduction initiatives. In this context, the widespread presence of iodized salt in the market does not necessarily guarantee adequate iodine intake across the population. Systematic monitoring of iodine concentrations in commercially available salt products may therefore represent an important complement to population-based iodine status surveillance.

From a clinical perspective, these findings also raise the possibility that insufficient iodine intake may persist in certain contexts. However, as the present study did not assess population iodine status or clinical outcomes, no direct conclusions can be drawn regarding the role of iodine intake in thyroid dysfunction. Nevertheless, iodine status may warrant consideration in selected clinical situations, particularly where dietary intake is uncertain. Further nationwide monitoring of iodine concentrations in salt products together with updated population iodine status surveys would help clarify the current effectiveness of iodine prophylaxis and support evidence-based public health and clinical decision-making.

## Figures and Tables

**Table 1:** Labelled characteristics of the purchased retail salt products.

Sample ID and Packaging	Declared Iodine Compound and Content (µg/100 g)	Declared Elemental Iodine Equivalent (ppm ^1^)	Country/Place of Origin	Manufacturer	Distributor	Best-Before Date
#1 Plastic bag 1 kg	KIO_3_ 1000	10	EU, Romania Transylvanian Salt Mines	Só-Center Packaging and Trade Social Cooperative, Nyírmada, Hungary	Reál Hungaria Food Ltd., Biatorbágy, Hungary	16 August 2027
#2 Paper box 1 kg	KIO_3_ 3000	30	Mediterranean Sea	Compagnia Italiana Sali S.p.a., Porto Viro, Italy	Hun-Trade Ltd., Budapest, Hungary	ND
#3 Plastic bag 1 kg	KIO_3_ 1000	10	EU, Romania Transylvanian Salt Mines	Só-Center Packaging and Trade Social Cooperative, Nyírmada, Hungary	CO-OP Hungary Zrt., Budapest, Hungary	11 December 2027
#4 Plastic bag 1 kg	KIO_3_ ND	ND	ND	ND	KOTÁNYI Hungaria Ltd., Budaörs, Hungary	5 January 2028
#5 Plastic bottle 0.5 kg	KIO_3_ 1180	11.8	EU, Turda, Romania, Salt Mine: Salina Valea Sarata	Laminoristilor str. 145F, Campia Turzii, Cluj, Romania	ND	17 November 2027
#6 Plastic bag 1 kg	KIO_3_ min. 20 mg/kg	11.9	EU	ND ^2^	Koch’s Torma (Hungaria) Ltd., Kiskunfélegyháza, Hungary	27 October 2027
#7 Plastic bag 1 kg	KIO_3_ 1180	11.8	Bosnia and Herzegovina	Solinwest 2000 Ltd. Tuzsér, Kálongatanya, Hungary info@solinwest.hu	ND ^2^	16 December 2027
#8 Plastic bag 1 kg	KIO_3_ 1500	15	ND ^2^	ND ^2^	METRO Trading Ltd., Budaörs, Hungary	26 January 2028
#9 Plastic bag 1 kg	KIO_3_ 2000	20	ND ^2^	Solinwest 2000 Ltd. Tuzsér, Kálongatanya, Hungary	ND ^2^	13 October 2027
#10 Plastic bag 1 kg	KIO_3_ 1000	10	EU, Romania Transylvanian Salt Mines	Só-Center Packaging and Trade Social Cooperative, Nyírmada, Hungary	ND ^2^	26 August 2027
#11 Plastic bag 1 kg	KI 15–25 mg/kg	11–19	Trinidad Sal Pans in the Ebro River Delta Nature Reserve on the Mediterranean coast of Spain	INFOSA, Tarragona, Spain	TONA Hungary Ltd., Gyál, Hungary	16 June 2028
#12 Plastic bottle 0.4 kg	ND iodine: 3700	37	Greece	KALAS S.A., Athens, Greece	EPIMPEX Ltd., Budapest, Hungary	28 August 2028
#13 Plastic bag 1 kg	KI 1500	15	Hallstatt, Austria	ND ^2^	Salinen Budapest Ltd., Budapest, Hungary	13 October 2028
#14 Plastic bag 1 kg	KI 3700	37	Greece	KALAS S.A., Athens, Greece	EPIMPEX Ltd., Budapest, Hungary	13 October 2028
#15 Plastic bag 1 kg	KI 1500	15	Austria	ND ^2^	Maresi Foodbroker Ltd., Budapest, Hungary	15 September 2028
#16 Plastic bottle 0.2 kg	KI (40–60 ppm) 5000 (75 ug/1.5 g) stabilizers: sodium carbonate (0.1%), magnesium carbonate (0.5%)	31–46	Greece	CHION S. A., Patras, Greece	Hellas-Invest Ltd., Törökbálint, Budapest	15 September 2030
#17 Plastic bag 1 kg	KI max. 1900	19	Wieliczka, Poland	ND ^2^	Kotányi Hungaria Ltd., Budapest, Hungary	August 2028
#18 Plastic bag 1 kg	KI 1500	15	Austria	ND ^2^	Maresi Foodbroker Ltd., Budapest, Hungary	15 December 2028
#19 Plastic bag 1 kg	KI 1500 stabilizer: sodium sulphate 0.2%	15	Romania	ND ^2^	Hadászi Ltd., Debrecen, Hungary	7 January 2028
#20 Plastic bag 1 kg	ND ^2^	ND ^2^	Pakistan	ND ^2^	VOG Export-Import Ltd., Bük, Hungary	Indefinite shelf life

^1^ ppm values represent the elemental iodine equivalent calculated from the declared iodine content where necessary. ^2^ ND: No data.

**Table 2:** Declared and measured iodine contents of the analyzed salt samples.

Sample ID and Packaging	Declared Iodine Compound	Declared Elemental Iodine Equivalent (ppm)	Mean Measured Elemental Iodine (I) Content in ppm (*n* = 3)	Relative Standard Deviation (RSD, %)
#1 Plastic bag 1 kg	KIO_3_	10	12.57	2.04
#2 Paper box 1 kg	KIO_3_	**30**	**29.32**	1.34
#3 Plastic bag 1 kg	KIO_3_	10	5.21	2.84
#4 Plastic bag 1 kg	KIO_3_	ND ^1^	17.95	1.43
#5 Plastic bottle 0.5 kg	KIO_3_	11.8	10.09	1.47
#6 Plastic bag 1 kg	KIO_3_	11.9	10.88	0.00
#7 Plastic bag 1 kg	KIO_3_	11.8	**21.16**	1.41
#8 Plastic bag 1 kg	KIO_3_	15	**21.25**	0.00
#9 Plastic bag 1 kg	KIO_3_	**20**	**22.80**	1.97
#10 Plastic bag 1 kg	KIO_3_	10	0	-
#11 Plastic bag 1 kg	KI	11–19	**28.34**	1.03
#12 Plastic bottle 0.4 kg	KI	**37**	17.94	0.82
#13 Plastic bag 1 kg	KI	15	16.50	1.54
#14 Plastic bag 1 kg	KI	**37**	**33.92**	0.86
#15 Plastic bag 1 kg	KI	15	9.98	1.47
#16 Plastic bottle 0.2 kg	KI	**31–46**	**30.20**	0.89
#17 Plastic bag 1 kg	KI	19	10.07	1.55
#18 Plastic bag 1 kg	KI	15	16.36	0.95
#19 Plastic bag 1 kg	KI	15	2.16	0
#20 Plastic bag 1 kg	KI	ND ^1^	5.30	2.94

^1^ ND: No data. Results are reported as the means of triplicate measurements with relative standard deviation (RSD, %). Bold values indicate measured iodine concentrations exceeding 20 ppm (lower limit of the WHO-recommended range for iodized salt).

**Table 3:** Distribution of measured iodine concentrations in commercially available iodized salts.

Iodine Concentration Category (ppm)	Samples (IDs)	Number (*n*)	Percentage
<10 ppm	#3, 10, 19, 20	4	20%
10–20 ppm	#1, 4, 5, 6, 12, 13, 15, 17, 18	9	45%
20–40 ppm	#2, 7, 8, 9, 11, 14, 16	7	35%
>40 ppm	–	0	0%
Total		20	100%

## Data Availability

The original contributions presented in this study are included in the article/Supplementary Material. Further inquiries can be directed to the corresponding author.

## Supplementary Materials

The following supporting information can be downloaded at https://www.mdpi.com/article/10.3390/nu18071164/s1, Table S1: Accuracy and linearity (KIO_3_), Table S2: Selectivity/matrix effect (KIO_3_), Table S3: Recovery (KIO_3_) (added 0.5 mL KIO_3_ stock solution, expected +5.0 mL titration volume factor = 1.00), Table S4: Accuracy and linearity (KI), Table S5: Selectivity/matrix effect (KI), Table S6: Recovery (KI) (added 0.2 mL KIO_3_ stock solution, expected +0.5 mL titration volume factor = 1.00).

## Abbreviations

The following abbreviations are used in this manuscript:

FT4	Free thyroxine
HACCP	Hazard Analysis and Critical Control Points
H_2_SO_4_	Sulfuric acid
IGN	Iodine Global Network
ISO	International Organization for Standardization
I_2_	Molecular iodine
I^−^	Iodide ion
KI	Potassium iodide
KIO_3_	Potassium iodate
LT4	Levothyroxine
NaCl	Sodium chloride
Na_2_S_2_O_3_	Sodium thiosulfate
NaOCl	Sodium hypochlorite
ND	No data
ppm	Parts per million
RSD	Relative standard deviation
TSH	Thyroid-stimulating hormone
UIC	Urinary iodine concentration
USI	Universal salt iodization
WHO	World Health Organization
