# Supplementary material for: Iodine Content of Commercially Available Iodized Salts in Hungary Determined by Iodometric Titration: Implications for the Effectiveness of Salt Iodization
Source: Nutrients. 2026 Apr 7;18(7):1164. doi: 10.3390/nu18071164 (PMC13074601; doi:10.3390/nu18071164)
Supplement: Supplementary file 1 [file nutrients-18-01164-s001.zip › nutrients-4226173-supplementary.pdf]

Validation (KIO_3)

The analytical method was validated by assessing accuracy and linearity across different iodine concentration ranges, followed by evaluation of precision through replicate measurements. Potential matrix effects were then examined using sodium chloride solutions to simulate the salt matrix, and recovery experiments were performed by spiking known amounts of potassium iodate (KIO_3) into commercial salt samples. Finally, reagent blank tests were conducted to verify the absence of background iodine formation. All measurements were performed in triplicate, and for each set of measurements the mean, standard deviation (SD), and relative standard deviation (RSD) were calculated.

Accuracy and linearity (Table S1)**:** To evaluate analytical accuracy across different concentration ranges, aliquots of 0.250 mL, 0.500 mL, 1.000 mL, and 2.000 mL of the KIO_3 stock solution were added to 200 mL of bidistilled water. Subsequently, 0.5 g of solid KI and 5 mL of 1 M sulfuric acid were added, the mixture was stored in the dark for 5 min, and the liberated iodine was titrated as described above.

Table S1. Accuracy and linearity (KIO_3)

Added KIO3 (1.7835 mg/ml) (ml)	Tiosulphate factor	Expected titration volume (factor=1.00) (ml)	Expected titration volume (ml)	Replicate 1 (ml)	Replicate 2 (ml)	Replicate 3 (ml)	Mean (ml)	SD	RSD (%)	Recovery % (Measured/Expected x100)
0.250	1.01	2.5	2.5	2.4	2.5	2.5	2.47	0.06	2.34%	99.65%
0.500	1.01	5.0	5.0	5.0	5.1	5.0	5.03	0.06	1.15%	101.67%
1.000	1.01	10.0	9.9	9.8	10.0	10.0	9.93	0.12	1.16%	100.33%
2.000	1.01	20.0	19.8	19.9	19.8	19.7	19.80	0.10	0.51%	99.99%

Precision: All measurements were performed in triplicate. Variability was low.

Selectivity/matrix effect (Table S2)**:** The same series of measurements (different concentration ranges) was then repeated in the presence of dissolved sodium chloride to simulate the sample matrix. For this purpose, 40 g of pharmaceutical-grade NaCl was dissolved in 200 mL of bidistilled water, after which the same volumes of KIO_3 stock solution were added with continuous stirring as in accuracy test. In the presence of NaCl, the recoverability of KIO_3 remained within 0.1–0.2 mL of titrant consumption compared with the mean values obtained in the absence of salt.

Table S2. Selectivity/matrix effect (KIO_3)

Added KIO3 (1.7835 mg/ml) (ml)	Tiosulphate factor	Expected titration volume (factor=1.00) (ml)	Expected titration volume (ml)	Replicate 1 (ml)	Replicate 2 (ml)	Replicate 3 (ml)	Mean (ml)	SD	RSD (%)	Recovery % (Measured/Expected x100)
0.250	1.01	2.5	2.5	2.4	2.4	2.3	2.37	0.06	2.44%	95.61%
0.500	1.01	5.0	5.0	5.0	4.9	4.8	4.90	0.10	2.04%	98.98%
1.000	1.01	10.0	9.7	9.8	9.8	10.0	9.87	0.12	1.17%	101.72%
2.000	1.01	20.0	19.8	19.7	19.6	19.7	19.67	0.06	0.29%	99.32%

Recovery (Table S3)**:** To assess potential matrix effects from commercial table salts, the actual salt samples were first analyzed for their intrinsic KIO_3 content. Subsequently, in the same experimental setup, 0.5 mL of the KIO_3 stock solution was added to the sample solutions to test whether naturally occurring or added substances in the salts could influence recovery. This experiment was performed for three different iodate-fortified salts. In all tested cases, the addition of KIO_3 produced an increase of approximately 5 mL in sodium thiosulfate consumption, consistent with the theoretically expected value. Similar results were obtained when KIO_3 was added to pure NaCl solutions, indicating good recovery in the salt matrix.

Table S3. Recovery (KIO_3) (added 0.5 mL KIO_3 stock solution, expected +5.0 mL titration volume factor=1.00)

Salt	Tiosulphate factor	Original titration volume (ml)	Replicate 1 (ml)	Replicate 2 (ml)	Replicate 3 (ml)	Mean (ml)	SD	RSD (%)		Recovery % (Measured - original/Expected x100)
#2	0.97	11.43	16.5	16.6	16.6	16.6	0.06	0.35%	5.15	99.65%
#5	0.97	3.93	9.1	9.0	9.0	9.0	0.06	0.64%	5.15	99.00%
#7	0.98	8.17	13.3	13.2	13.2	13.2	0.06	0.44%	5.10	99.24%

Blank test: Blank experiments were also performed. In these tests, 0.5 g KI and 5 mL of 1 M sulfuric acid were added to 200 mL of bidistilled water. The mixture was kept in a closed flask in the dark for 5 min, after which 1 mL of 0.8% starch solution was added and titration with sodium thiosulfate was initiated. The solution was monitored for the appearance of a visible blue color up to a titrant consumption of 20 mL. No color change was observed, indicating the absence of detectable background iodine.

Validation (KI)

Accuracy and linearity (Table S4): To evaluate analytical accuracy across different concentration ranges, aliquots of 0.200 mL, 0.500 mL, 0.700 mL of the KI stock solution were added to 300 mL of bidistilled water. Subsequently, 7 mL of sulfuric acid and 1 mL of 5% sodium hypochlorite solution ("hypo") were added. To ensure completion of the reaction, the solution was stirred for 5 min and the mixture was stirred, boiled, and then allowed to cool. Based on pH measurements, the solution had a pH of approximately 2 (or slightly below), and therefore no additional acidification was considered necessary. After cooling, 0.5 g of solid KI was added under continuous stirring, and the solution was kept in a closed container in the dark for 5 min, and the liberated iodine was titrated as described above.

Table S4. Accuracy and linearity (KI)

Added KIO_3 (1.7835 mg/ml) (ml)	Tiosulphate factor	Expected titration volume (factor=1.00) (ml)	Expected titration volume (ml)	Replicate 1 (ml)	Replicate 2 (ml)	Replicate 3 (ml)	Mean (ml)	SD	RSD (%)	Recovery % (Measured/Expected x100)
0.200	1.01	5.0	5.0	4.9	5.0	5.0	4.97	0.06	1.16%	100.33%
0.500	1.01	12.5	12.4	12.5	12.4	12.4	12.43	0.06	0.46%	100.46%
0.700	1.01	17.5	17.3	17.3	17.2	17.2	17.23	0.06	0.34%	99.46%

Precision: All measurements were performed in triplicate. Variability was low.

Selectivity/matrix effect (Table S5)**:** The same series of measurements (different concentration ranges) was then repeated in the presence of dissolved sodium chloride to simulate the sample matrix. For this purpose, 40 g of pharmaceutical-grade NaCl was dissolved in 300 mL of bidistilled water, after which the same volumes of KI stock solution were added with continuous stirring as in accuracy test. In the presence of NaCl, the recoverability of KI remained within 0.1–0.2 mL of titrant consumption compared with the mean values obtained in the absence of salt.

Table S5. Selectivity/matrix effect (KI)

Added KIO_3 (1.7835 mg/ml) (ml)	Tiosulphate factor	Expected titration volume (factor=1.00) (ml)	Expected titration volume (ml)	Replicate 1 (ml)	Replicate 2 (ml)	Replicate 3 (ml)	Mean (ml)	SD	RSD (%)	Recovery % (Measured/Expected x100)
0.200	1.01	5.0	5.0	4.9	4.9	4.9	4.90	0.00	0.00%	98.98%
0.500	1.01	12.5	12.4	12.4	12.3	12.3	12.33	0.06	0.47%	99.65%
0.700	1.01	17.5	17.3	17.3	17.4	17.4	17.37	0.06	0.33%	100.23%

Recovery (Table S6)**:** To assess potential matrix effects from commercial table salts, the actual salt samples were first analyzed for their intrinsic KIO_3 content. Subsequently, in the same experimental setup, 0.2 mL of the KI stock solution was added to the sample solutions to test whether naturally occurring or added substances in the salts could influence recovery. This experiment was performed for three different iodate-fortified salts. In all tested cases, the addition of KI produced an increase of approximately 5 mL in sodium thiosulfate consumption, consistent with the theoretically expected value. Similar results were obtained when KI was added to pure NaCl solutions, indicating good recovery in the salt matrix.

Table S6. Recovery (KI) (added 0.2 mL KIO_3 stock solution, expected +0.5 mL titration volume factor=1.00)

Salt	Tiosulphate factor	Original titration volume (ml)	Replicate 1 (ml)	Replicate 2 (ml)	Replicate 3 (ml)	Mean (ml)	SD	RSD (%)		Recovery % (Measured - original/Expected x100)
#11	0.96	11.17	16.4	16.4	16.4	16.4	0.00	0.00%	5.21	100.42%
#12	0.96	7.07	12.2	12.1	12.3	12.2	0.10	0.82%	5.21	98.50%
#16	1.02	11.20	16.0	16.2	16.1	16.1	0.10	0.62%	4.90	99.96%

Blank test: Blank experiments were also performed. In these tests, 300 mL of bidistilled water was used. Subsequently, 7 mL of sulfuric acid and 1 mL of 5% sodium hypochlorite solution ("hypo") were added. To ensure completion of the reaction, the solution was stirred for 5 min and the mixture was stirred, boiled, and then allowed to cool. Based on pH measurements, the solution had a pH of approximately 2 (or slightly below), and therefore no additional acidification was considered necessary. After cooling, 0.5 g of solid KI was added under continuous stirring, and the solution was kept in a closed container in the dark for 5 min, after which 1 mL of 0.8% starch solution was added and titration with sodium thiosulfate was initiated. The solution was monitored for the appearance of a visible blue color up to a titrant consumption of 20 mL. No color change was observed, indicating the absence of detectable background iodine.
